# Supplementary material for: New Insights on the PBMCs Phospholipidome in Obesity Demonstrate Modulations Associated with Insulin Resistance and Glycemic Status
Source: Nutrients. 2021 Sep 29;13(10):3461. doi: 10.3390/nu13103461 (PMC8541295; doi:10.3390/nu13103461)
Supplement: Supplementary file 1 [file nutrients-13-03461-s001.zip › nutrients-1371910-supplementary.pdf]

SUPPLEMENTARY MATERIALS:

New insights on the PBMCs phospholipidome in obesity demonstrate modulations associated with insulin resistance and glycemic status

Chloé Wilkin[1], Megan Colonval[1], Jonas Dehairs[2], Nathalie Esser[1,3], Margaud Iovino[1], Marco A. Gianfrancesco[1,3], Marjorie Fadeur[1,3], Johan V. Swinnen[2], Nicolas Paquot[1,3], Jacques Piette[4] and Sylvie Legrand-Poels[1,*]

[1] Laboratory of Immunometabolism and Nutrition, GIGA-Inflammation, Infection & Immunity, University of Liège, Liège, Belgium
[2] Laboratory of Lipid Metabolism and Cancer, Department of Oncology, KU Leuven, Leuven, Belgium
[3] Division of Diabetes, Nutrition and Metabolic Disorders, Department of Medicine, University Hospital of Liège, Liège, Belgium
[4] Laboratory of Virology and Immunology, GIGA-Molecular Biology of Diseases, University of Liège, Liège, Belgium
[*] Correspondence: s.legrand@uliege.be, Tel: +32 4 366 24 34, Immunometabolism and Nutrition (GIGA), Avenue de l'Hôpital1/11, B34, 4000 Liège, Belgium

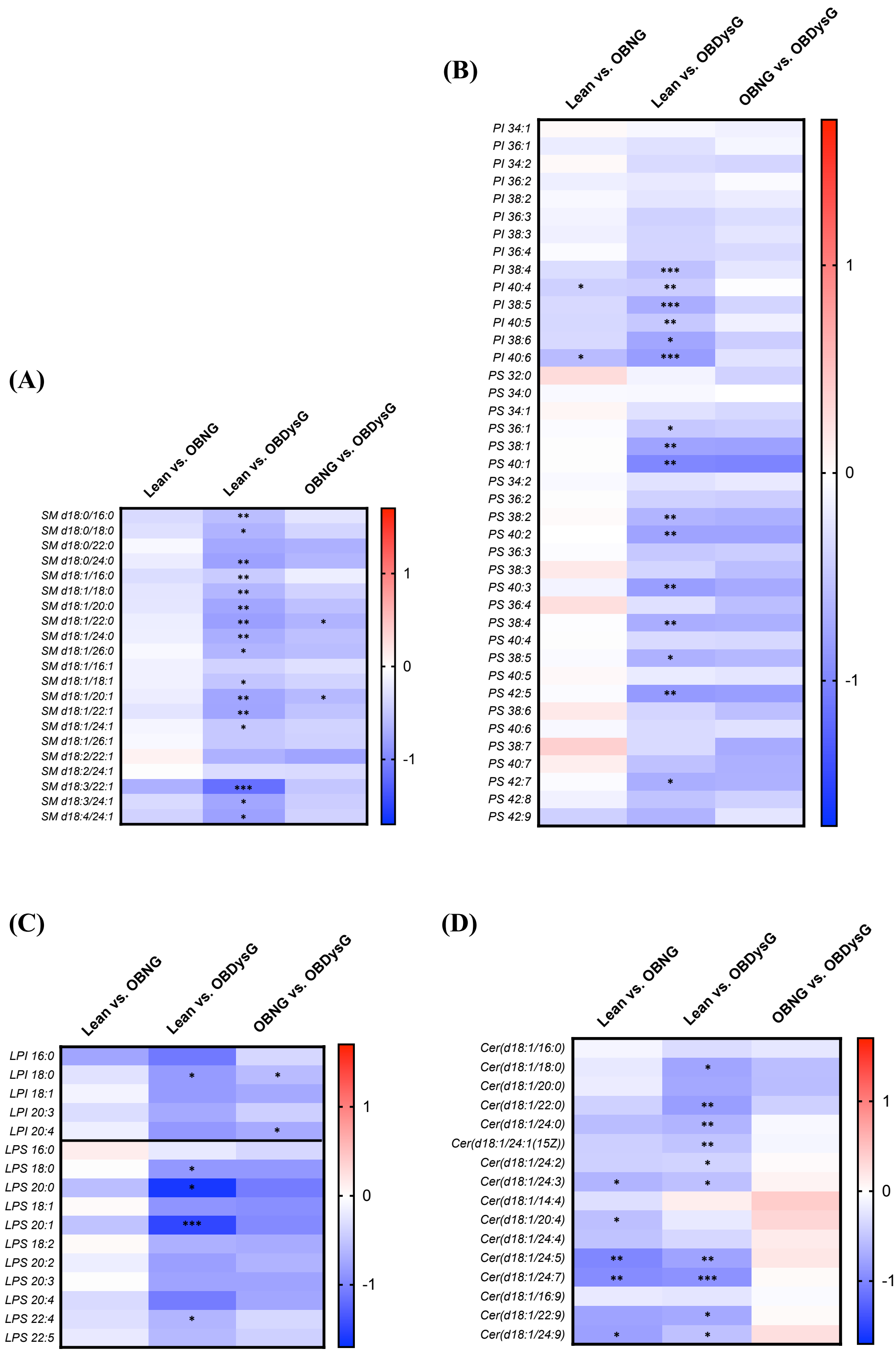

Figure S1. Fold change (Log2FC) of all (A) SM, (B) PI/PS, (C) Lyso-PI/-PS and (D) Cer lipid species detected in PBMCs from lean compared to OBNG or OBDysG patients and from OBNG vs. OBDysG individuals. Mean comparison * p≤ 0.05; **p ≤ 0.01; ***p ≤ 0.001. Kruskal-Wallis followed by Dunn post hoc test was performed on data.

Table S1. Participants blood cells composition. Data are mean ± SD. Kruskal-Wallis followed by Dunn post hoc test was performed on data. OBNG or OBIG *vs.* Lean * p≤ 0.05; **p ≤ 0.01; ***p ≤ 0.001. OBIG *vs.* OBNG †p≤ 0.05; ††p ≤ 0.01; †††p ≤ 0.001.

	Overall p-value	Lean	OBNG	OBDysG
Platelets ($10^3/mm^3$)	0.238	244.6 ± 37.7	293.7 ± 104.0	255.6 ± 50.0
Leucocytes ($10^3/mm^3$)	0.004	6.1 ± 1.2	7.7 ± 2.0 *	8.0 ± 1.8 **
Neutrophils (%)	0.375	56.8 ± 9.2	56.8 ± 6.0	59.2 ± 7.1
Lymphocytes (%)	0.22	32.5 ± 7.2	33.2 ± 6.4	29.5 ± 6.6
Monocytes (%)	0.275	7.94 ± 1.39	7.07 ± 1.79	7.59 ± 2.14
Eosinophils (%)	0.216	2.27 ± 1.65	2.27 ± 1.40	2.82 ± 1.38
Basophiles (%)	0.869	0.48 ± 0.22	0.54 ± 0.36	0.52 ± 0.22
Neutrophils absolute ($10^3/mm^3$)	0.024	3.53 ± 1.19	4.36 ± 1.32	4.79 ± 1.49 *
Lymphocytes absolute ($10^3/mm^3$)	0.012	1.92 ± 0.36	2.55 ± 0.76 **	2.32 ± 0.65
Monocytes absolute ($10^3/mm^3$)	0.096	0.47 ± 0.09	0.54 ± 0.19	0.60 ± 0.18
Eosinophils absolute ($10^3/mm^3$)	0.017	0.13 ± 0.09	0.17 ± 0.12	0.21 ± 0.12 *
Basophiles absolute ($10^3/mm^3$)	0.102	0.029 ± 0.013	0.040 ± 0.027	0.255 ± 1.035

Table S2. Individual PBMCs lipid species. Data are mean ± SD.

Lipid species (nmol/mg DNA)	Lean	OBNG	OBIG
PC 28:0	0.143 ± 0.062	0.13 ± 0.073	0.08 ± 0.057
PC 30:0	2.882 ± 0.886	2.299 ± 0.931	1.845 ± 0.742
PC 32:0	61.659 ± 13.396	50.188 ± 13.016	46.932 ± 14.065
PC 34:0	13.566 ± 3.539	10.416 ± 3.361	8.831 ± 3.513
PC 36:0	2.133 ± 0.765	1.667 ± 0.707	1.277 ± 0.724
PC 38:0	1.882 ± 0.422	1.476 ± 0.512	1.437 ± 0.621
PC 40:0	0.307 ± 0.152	0.232 ± 0.153	0.163 ± 0.116
PC 32:1	6.713 ± 1.81	6.242 ± 2.272	4.921 ± 1.835
PC 34:1	143.536 ± 41.405	118.489 ± 40.52	95.26 ± 35.32
PC 36:1	49.229 ± 13.757	39.824 ± 14.686	30.659 ± 13.046
PC 40:1	0.421 ± 0.118	0.346 ± 0.13	0.263 ± 0.103
PC 32:2	0.655 ± 0.213	0.522 ± 0.283	0.35 ± 0.161
PC 34:2	50.128 ± 13.158	37.071 ± 12.733	29.893 ± 12.119
PC 36:2	62.639 ± 11.081	49.946 ± 12.957	42.966 ± 14.997
PC 38:2	4.763 ± 1.25	3.321 ± 1.348	2.564 ± 1.272
PC 40:2	0.459 ± 0.148	0.376 ± 0.153	0.285 ± 0.119
PC 42:2	0.345 ± 0.099	0.29 ± 0.113	0.225 ± 0.088
PC 44:2	0.354 ± 0.226	0.282 ± 0.111	0.222 ± 0.122
PC 32:3	0.182 ± 0.079	0.138 ± 0.056	0.111 ± 0.058
PC 34:3	2.515 ± 0.618	2.097 ± 0.863	1.511 ± 0.637
PC 36:3	32.44 ± 5.272	26.618 ± 7.242	21.573 ± 7.474
PC 38:3	13.762 ± 2.533	12.099 ± 3.798	9.096 ± 3.272
PC 40:3	0.789 ± 0.214	0.702 ± 0.306	0.498 ± 0.189
PC 42:3	0.258 ± 0.09	0.237 ± 0.074	0.183 ± 0.079
PC 44:3	0.28 ± 0.187	0.227 ± 0.095	0.174 ± 0.105
PC 34:4	0.824 ± 0.282	0.663 ± 0.312	0.474 ± 0.243
PC 36:4	65.946 ± 15.286	54.066 ± 16.761	46.74 ± 15.803
PC 38:4	77.49 ± 17.416	64.194 ± 17.667	56.402 ± 20.47
PC 40:4	6.039 ± 1.717	5.151 ± 1.923	3.965 ± 1.699
PC 42:4	0.503 ± 0.152	0.433 ± 0.169	0.318 ± 0.14
PC 44:4	0.2 ± 0.152	0.179 ± 0.08	0.128 ± 0.069
PC 36:5	3.641 ± 0.935	3.088 ± 1.121	2.501 ± 1.045
PC 38:5	36.565 ± 5.891	30.847 ± 8.617	26.964 ± 9.185
PC 40:5	10.59 ± 2.413	9.141 ± 3.082	7.745 ± 2.921
PC 42:5	0.869 ± 0.235	0.741 ± 0.304	0.583 ± 0.243
PC 44:5	0.399 ± 0.219	0.317 ± 0.128	0.243 ± 0.133
PC 36:6	0.39 ± 0.175	0.334 ± 0.16	0.25 ± 0.135
PC 38:6	11.553 ± 2.458	8.6 ± 2.65	7.462 ± 2.647

Lipid species (nmol/mg DNA)	Lean	OBNG	OBIG
PC 40:6	9.497 ± 1.969	7.518 ± 2.291	6.579 ± 2.247
PC 42:6	1.032 ± 0.249	0.86 ± 0.334	0.673 ± 0.262
PC 44:6	0.469 ± 0.218	0.362 ± 0.184	0.267 ± 0.157
PC 38:7	1.002 ± 0.299	0.842 ± 0.311	0.642 ± 0.276
PC 40:7	4.903 ± 1.337	4.082 ± 1.567	3.171 ± 1.295
PC 42:7	1.167 ± 0.291	0.985 ± 0.374	0.771 ± 0.279
PC 44:7	0.319 ± 0.151	0.256 ± 0.113	0.198 ± 0.1
PC 40:8	2.403 ± 0.7	2.023 ± 0.863	1.56 ± 0.717
PC 42:8	1.336 ± 0.388	1.144 ± 0.514	0.876 ± 0.369
PC 44:8	0.437 ± 0.166	0.418 ± 0.153	0.346 ± 0.149
PC 42:9	1.031 ± 0.306	0.964 ± 0.397	0.753 ± 0.379
PC 44:9	0.367 ± 0.123	0.338 ± 0.122	0.255 ± 0.111
PC 42:10	0.953 ± 0.281	0.854 ± 0.323	0.7 ± 0.374
PC 44:10	0.47 ± 0.147	0.396 ± 0.151	0.311 ± 0.119
PC 42:11	0.898 ± 0.256	0.812 ± 0.29	0.658 ± 0.318
PC 44:11	0.748 ± 0.259	0.681 ± 0.238	0.557 ± 0.28
PC 42:12	1.36 ± 0.431	1.179 ± 0.401	1.028 ± 0.469
**PC - total**	695.439 ± 139.616	566.7 ± 166.05	474.443 ± 161.902
SM d18:0/16:0	1.887 ± 0.539	1.526 ± 0.546	1.295 ± 0.402
SM d18:0/18:0	0.539 ± 0.21	0.447 ± 0.221	0.347 ± 0.164
SM d18:0/22:0	2.093 ± 1.181	2.002 ± 1.342	1.267 ± 0.764
SM d18:0/24:0	1.051 ± 0.442	0.938 ± 0.479	0.616 ± 0.307
SM d18:1/16:0	29.992 ± 6.904	24.492 ± 6.379	22.086 ± 7.412
SM d18:1/18:0	2.391 ± 0.766	2.041 ± 0.715	1.565 ± 0.707
SM d18:1/20:0	5.047 ± 1.881	4.342 ± 2.236	3.027 ± 1.517
SM d18:1/22:0	15.339 ± 6.138	13.837 ± 7.397	8.886 ± 4.51
SM d18:1/24:0	11.271 ± 3.869	10.233 ± 4.429	7.087 ± 3.257
SM d18:1/26:0	0.243 ± 0.088	0.233 ± 0.109	0.158 ± 0.075
SM d18:1/16:1	1.506 ± 0.479	1.386 ± 0.471	1.154 ± 0.458
SM d18:1/18:1	0.636 ± 0.209	0.587 ± 0.199	0.453 ± 0.206
SM d18:1/20:1	0.683 ± 0.229	0.614 ± 0.254	0.41 ± 0.198
SM d18:1/22:1	6.084 ± 1.983	5.168 ± 2.458	3.63 ± 1.628
SM d18:1/24:1	23.002 ± 6.985	21.629 ± 8.308	16.664 ± 6.955
SM d18:1/26:1	0.514 ± 0.162	0.492 ± 0.195	0.374 ± 0.153
SM d18:2/22:1	1.533 ± 0.989	1.65 ± 1.245	0.981 ± 0.633
SM d18:2/24:1	5.907 ± 1.733	5.951 ± 2.158	4.785 ± 1.965
SM d18:3/22:1	1.08 ± 0.509	0.687 ± 0.424	0.492 ± 0.288
SM d18:3/24:1	1.891 ± 0.725	1.524 ± 0.76	1.142 ± 0.721
SM d18:4/24:1	0.744 ± 0.346	0.571 ± 0.334	0.437 ± 0.242

Lipid species (nmol/mg DNA)	Lean	OBNG	OBIG
**SM - total**	113.433 ± 32.453	100.349 ± 38.239	76.856 ± 30.385
PE 32:0	0.274 ± 0.093	0.209 ± 0.065	0.206 ± 0.062
PE 34:0	0.09 ± 0.058	0.068 ± 0.039	0.085 ± 0.038
PE 36:0	0.224 ± 0.133	0.23 ± 0.139	0.242 ± 0.135
PE 38:0	0.469 ± 0.169	0.426 ± 0.165	0.44 ± 0.192
PE 40:0	0.125 ± 0.046	0.105 ± 0.049	0.085 ± 0.037
PE 26:1	0.574 ± 1.176	0.21 ± 0.163	0.187 ± 0.304
PE 28:1	0.396 ± 0.827	0.137 ± 0.111	0.111 ± 0.16
PE 32:1	0.156 ± 0.059	0.145 ± 0.058	0.122 ± 0.051
PE 34:1	5.754 ± 2.254	4.503 ± 1.55	3.813 ± 1.386
PE 36:1	10.731 ± 2.062	8.492 ± 2.544	8.215 ± 2.627
PE 38:1	1.296 ± 0.755	1.128 ± 0.565	1.054 ± 0.55
PE 40:1	0.301 ± 0.171	0.27 ± 0.133	0.235 ± 0.131
PE 36:2	11.339 ± 2.998	8.626 ± 2.492	7.45 ± 2.79
PE 38:2	1.774 ± 0.68	1.423 ± 0.553	1.23 ± 0.491
PE 40:2	0.353 ± 0.145	0.308 ± 0.124	0.292 ± 0.139
PE 42:2	0.077 ± 0.037	0.061 ± 0.022	0.058 ± 0.025
PE 34:3	0.112 ± 0.035	0.1 ± 0.042	0.094 ± 0.036
PE 36:3	3.291 ± 1.062	2.546 ± 0.902	2.051 ± 0.891
PE 38:3	5.065 ± 0.862	4.774 ± 1.501	3.825 ± 1.17
PE 40:3	0.369 ± 0.102	0.291 ± 0.135	0.26 ± 0.104
PE 42:3	0.054 ± 0.016	0.041 ± 0.02	0.037 ± 0.019
PE 36:4	7.528 ± 2.467	5.901 ± 2.573	4.748 ± 1.943
PE 38:4	53.687 ± 10.847	43.909 ± 14.386	37.292 ± 12.511
PE 40:4	6.675 ± 1.757	5.505 ± 1.765	4.623 ± 1.761
PE 42:4	0.203 ± 0.05	0.181 ± 0.072	0.131 ± 0.05
PE 36:5	0.427 ± 0.124	0.348 ± 0.125	0.297 ± 0.115
PE 38:5	12.397 ± 3.527	9.752 ± 3.756	7.797 ± 3.088
PE 40:5	7.785 ± 1.43	6.713 ± 1.889	5.862 ± 1.964
PE 42:5	0.356 ± 0.096	0.299 ± 0.123	0.219 ± 0.084
PE 38:6	3.29 ± 0.944	2.439 ± 0.841	2.111 ± 0.809
PE 40:6	6.563 ± 1.233	5.362 ± 1.581	4.892 ± 1.677
PE 42:6	0.321 ± 0.093	0.272 ± 0.113	0.198 ± 0.075
PE 38:7	1.303 ± 0.441	1.011 ± 0.417	0.813 ± 0.327
PE 40:7	10.991 ± 2.636	9.071 ± 3.276	7.494 ± 2.688
PE 42:7	0.799 ± 0.224	0.671 ± 0.245	0.531 ± 0.208
PE 40:8	1.866 ± 0.53	1.479 ± 0.631	1.175 ± 0.477
PE 42:8	0.733 ± 0.174	0.639 ± 0.24	0.54 ± 0.206
PE 42:9	0.933 ± 0.243	0.758 ± 0.283	0.67 ± 0.24

Lipid species (nmol/mg DNA)	Lean	OBNG	OBIG
PE 42:10	0.32 ± 0.101	0.248 ± 0.108	0.207 ± 0.086
PE 42:11	0.147 ± 0.049	0.11 ± 0.052	0.092 ± 0.047
PE 44:11	0.061 ± 0.019	0.05 ± 0.03	0.038 ± 0.027
PE 42:12	0.204 ± 0.072	0.175 ± 0.061	0.154 ± 0.072
**PE - total**	159.412 ± 32.413	128.985 ± 40.019	109.981 ± 36.226
PI 34:1	0.912 ± 0.583	0.945 ± 0.361	0.87 ± 0.348
PI 36:1	2.199 ± 1.233	1.954 ± 0.648	1.844 ± 0.739
PI 34:2	0.441 ± 0.296	0.456 ± 0.19	0.356 ± 0.15
PI 36:2	3.289 ± 1.24	2.99 ± 0.962	2.895 ± 1.036
PI 38:2	0.232 ± 0.115	0.222 ± 0.1	0.198 ± 0.119
PI 36:3	0.886 ± 0.346	0.828 ± 0.329	0.674 ± 0.22
PI 38:3	4.755 ± 1.298	4.342 ± 1.399	3.704 ± 1.162
PI 36:4	3.028 ± 0.851	2.956 ± 1.297	2.377 ± 0.754
PI 38:4	60.881 ± 11.41	49.586 ± 17.554	42.608 ± 13.404
PI 40:4	1.132 ± 0.207	0.855 ± 0.257	0.843 ± 0.292
PI 38:5	5.652 ± 1.464	4.523 ± 2.132	3.513 ± 1.292
PI 40:5	1.262 ± 0.183	0.998 ± 0.438	0.911 ± 0.308
PI 38:6	0.315 ± 0.153	0.252 ± 0.171	0.189 ± 0.09
PI 40:6	0.58 ± 0.196	0.391 ± 0.184	0.331 ± 0.112
**PI - total**	85.561 ± 15.651	71.298 ± 24.366	61.313 ± 18.898
PS 32:0	0.122 ± 0.036	0.148 ± 0.162	0.114 ± 0.054
PS 34:0	0.46 ± 0.141	0.443 ± 0.252	0.441 ± 0.197
PS 34:1	1.565 ± 0.344	1.651 ± 1.086	1.309 ± 0.469
PS 36:1	49.634 ± 15.37	48.357 ± 32.515	35.849 ± 14.231
PS 38:1	2.065 ± 0.771	2.087 ± 1.621	1.221 ± 0.593
PS 40:1	0.632 ± 0.27	0.638 ± 0.624	0.322 ± 0.19
PS 34:2	0.168 ± 0.04	0.161 ± 0.062	0.142 ± 0.056
PS 36:2	7.008 ± 1.839	7.081 ± 4.446	5.312 ± 2.04
PS 38:2	2.305 ± 0.788	2.37 ± 1.726	1.494 ± 0.548
PS 40:2	0.809 ± 0.331	0.809 ± 0.67	0.478 ± 0.21
PS 36:3	0.552 ± 0.233	0.539 ± 0.306	0.4 ± 0.155
PS 38:3	9.18 ± 3.136	10.447 ± 7.02	7.138 ± 2.497
PS 40:3	0.29 ± 0.107	0.271 ± 0.168	0.166 ± 0.074
PS 36:4	0.365 ± 0.109	0.442 ± 0.367	0.303 ± 0.12
PS 38:4	34.541 ± 11.735	34.161 ± 25.209	21.523 ± 10.938
PS 40:4	5.644 ± 1.645	5.713 ± 3.424	4.531 ± 1.779
PS 38:5	2.119 ± 0.742	2.057 ± 1.495	1.358 ± 0.656
PS 40:5	6.895 ± 1.869	7.157 ± 3.511	6.094 ± 2.087
PS 42:5	0.27 ± 0.106	0.263 ± 0.198	0.152 ± 0.053

Lipid species (nmol/mg DNA)	Lean	OBNG	OBIG
PS 38:6	0.244 ± 0.056	0.278 ± 0.22	0.191 ± 0.086
PS 40:6	6.329 ± 1.758	6.065 ± 3.068	5.07 ± 2.019
PS 38:7	0.019 ± 0.028	0.025 ± 0.04	0.015 ± 0.014
PS 40:7	0.77 ± 0.345	0.849 ± 0.689	0.538 ± 0.27
PS 42:7	0.118 ± 0.048	0.116 ± 0.062	0.074 ± 0.029
PS 42:8	0.134 ± 0.091	0.124 ± 0.089	0.094 ± 0.046
PS 42:9	0.18 ± 0.143	0.136 ± 0.066	0.116 ± 0.077
**PS - total**	132.415 ± 35.789	132.388 ± 86.728	94.445 ± 35.832
Cer(d18:1/16:0)	0.0246 ± 0.0061	0.0230 ± 0.0066	0.0199 ± 0.0076
Cer(d18:1/18:0)	0.0039 ± 0.0023	0.0034 ± 0.0021	0.0024 ± 0.0012
Cer(d18:1/20:0)	0.0066 ± 0.0035	0.0059 ± 0.0041	0.0038 ± 0.0022
Cer(d18:1/22:0)	0.0174 ± 0.0077	0.0132 ± 0.0075	0.0100 ± 0.0059
Cer(d18:1/24:0)	0.017 ± 0.0058	0.0115 ± 0.0046	0.0110 ± 0.0067
Cer(d18:1/24:1(15Z))	0.0179 ± 0.005	0.0134 ± 0.0044	0.0127 ± 0.0041
Cer(d18:1/24:2)	0.0018 ± 0.0004	0.0013 ± 0.0005	0.0014 ± 0.0006
Cer(d18:1/24:3)	0.0013 ± 0.0005	0.0009 ± 0.0004	0.0009 ± 0.0005
Cer(d18:1/14:4)	0.0001 ± 0.0001	0.0001 ± 0.0001	0.0001 ± 0.0001
Cer(d18:1/20:4)	0.0003 ± 0.0001	0.0002 ± 0.0001	0.0003 ± 0.0002
Cer(d18:1/24:4)	0.0004 ± 0.0002	0.0003 ± 0.0002	0.0003 ± 0.0004
Cer(d18:1/24:5)	0.0007 ± 0.0003	0.0004 ± 0.0003	0.0004 ± 0.0011
Cer(d18:1/24:7)	0.0028 ± 0.0011	0.0015 ± 0.0007	0.0015 ± 0.0007
Cer(d18:1/16:9)	0.0019 ± 0.0009	0.0016 ± 0.0007	0.0020 ± 0.0008
Cer(d18:1/22:9)	0.0013 ± 0.0007	0.0008 ± 0.0004	0.0008 ± 0.0008
Cer(d18:1/24:9)	0.0013 ± 0.0006	0.0008 ± 0.0004	0.0009 ± 0.0007
**Cer - total**	0.099 ± 0.03	0.078 ± 0.029	0.068 ± 0.027
LPC 14:0	0.209 ± 0.103	0.159 ± 0.085	0.104 ± 0.06
LPC 16:0	24.589 ± 11.782	18.543 ± 8.17	13.798 ± 5.938
LPC 18:0	13.248 ± 6.046	9.63 ± 3.984	7.201 ± 3.152
LPC 20:0	0.796 ± 0.322	0.62 ± 0.339	0.383 ± 0.243
LPC 22:0	0.489 ± 0.489	0.403 ± 0.315	0.255 ± 0.251
LPC 16:1	0.758 ± 0.44	0.674 ± 0.34	0.441 ± 0.204
LPC 18:1	23.59 ± 12.172	18.39 ± 8.623	12.854 ± 5.744
LPC 20:1	1.258 ± 0.579	0.943 ± 0.427	0.627 ± 0.307
LPC 22:1	0.474 ± 0.419	0.41 ± 0.298	0.267 ± 0.24
LPC 16:2	0.038 ± 0.02	0.039 ± 0.022	0.027 ± 0.014
LPC 18:2	9.793 ± 5.272	6.805 ± 2.88	4.928 ± 2.411
LPC 20:2	1.019 ± 0.566	0.765 ± 0.308	0.589 ± 0.267
LPC 22:2	0.432 ± 0.486	0.379 ± 0.321	0.252 ± 0.267
LPC 18:3	0.217 ± 0.112	0.177 ± 0.093	0.109 ± 0.057

Lipid species (nmol/mg DNA)	Lean	OBNG	OBIG
LPC 20:3	2.716 ± 1.504	2.226 ± 0.925	1.509 ± 0.635
LPC 22:3	0.536 ± 0.543	0.486 ± 0.371	0.319 ± 0.291
LPC 20:4	17.78 ± 8.975	12.787 ± 5.67	9.613 ± 4.925
LPC 22:4	1.461 ± 0.95	1.179 ± 0.638	0.808 ± 0.553
LPC 22:5	1.781 ± 1.169	1.367 ± 0.797	1.005 ± 0.685
**LPC - total**	101.184 ± 49.085	75.983 ± 32.592	55.091 ± 24.725
LPE 16:0	1.339 ± 0.555	0.978 ± 0.539	0.627 ± 0.436
LPE 18:0	5.685 ± 2.546	4.648 ± 2.117	3.275 ± 1.708
LPE 16:1	0.21 ± 0.225	0.157 ± 0.137	0.089 ± 0.112
LPE 18:1	5.362 ± 3.405	4.072 ± 3.056	2.411 ± 2.18
LPE 20:1	0.313 ± 0.219	0.243 ± 0.175	0.139 ± 0.129
LPE 18:2	1.722 ± 1.114	1.223 ± 0.95	0.772 ± 0.858
LPE 20:2	0.231 ± 0.128	0.172 ± 0.101	0.099 ± 0.067
LPE 20:3	1.73 ± 1.994	1.142 ± 0.689	0.674 ± 0.865
LPE 22:3	2.029 ± 2.845	1.062 ± 0.73	0.637 ± 0.962
LPE 20:4	16.055 ± 13.963	10.594 ± 10.371	5.474 ± 8.506
LPE 22:4	9.928 ± 8.792	7.2 ± 7.895	3.621 ± 6.351
LPE 20:5	0.384 ± 0.315	0.314 ± 0.295	0.161 ± 0.204
LPE 22:5	8.14 ± 6.654	5.816 ± 6.357	3.136 ± 4.757
**LPE - total**	53.129 ± 39.071	37.621 ± 31.351	21.114 ± 25.871
LPI 16:0	0.23 ± 0.281	0.137 ± 0.168	0.109 ± 0.081
LPI 18:0	1.174 ± 0.665	0.991 ± 0.583	0.667 ± 0.235
LPI 18:1	0.156 ± 0.123	0.145 ± 0.094	0.088 ± 0.05
LPI 20:3	0.109 ± 0.07	0.089 ± 0.075	0.067 ± 0.039
LPI 20:4	1.062 ± 0.65	0.965 ± 0.657	0.592 ± 0.235
**LPI - total**	2.732 ± 1.643	2.328 ± 1.52	1.523 ± 0.54
LPS 16:0	0.107 ± 0.095	0.261 ± 0.612	0.094 ± 0.072
LPS 18:0	3.696 ± 2.111	3.703 ± 4.166	2.055 ± 0.71
LPS 20:0	0.092 ± 0.07	0.063 ± 0.077	0.031 ± 0.015
LPS 18:1	2.379 ± 1.382	2.446 ± 3.15	1.296 ± 0.483
LPS 20:1	0.065 ± 0.044	0.045 ± 0.049	0.024 ± 0.011
LPS 18:2	0.137 ± 0.103	0.142 ± 0.172	0.087 ± 0.038
LPS 20:2	0.031 ± 0.018	0.028 ± 0.025	0.018 ± 0.01
LPS 20:3	0.288 ± 0.177	0.288 ± 0.322	0.17 ± 0.074
LPS 20:4	0.9 ± 0.654	0.722 ± 0.632	0.435 ± 0.206
LPS 22:4	0.098 ± 0.043	0.081 ± 0.056	0.064 ± 0.024
LPS 22:5	0.116 ± 0.06	0.102 ± 0.075	0.077 ± 0.029
**LPS - total**	7.909 ± 4.583	7.881 ± 9.253	4.35 ± 1.502

Table S3. Relative abundance of each lipid class. Data are mean ± SD. Lean vs. OBNG or OBDysG * p≤ 0.05. Kruskal-Wallis followed by Dunn post hoc test was performed on data.

	**Overall p-value**	**Lean**	**OBNG**	**OBDysG**
% PC	0.188	51.60 ± 3.87	51.06 ± 3.44	52.97 ± 2.52
% SM	0.393	8.33 ± 1.38	8.89 ± 1.12	8.43 ± 0.88
% PE	0.105	11.90 ± 1.68	11.59 ± 1.03	12.33 ± 0.86
% PI	0.055	6.38 ± 0.66	6.38 ± 0.89	6.93 ± 0.84
% PS	0.414	9.76 ± 1.85	11.31 ± 4.08	10.48 ± 2.42
% Cer	0.33	0.007 ± 0.001	0.007 ± 0.001	0.008 ± 0.003
% LPC	0.245	7.46 ± 3.70	6.68 ± 1.29	5.99 ± 1.25
% LPE	0.012	3.80 ± 2.22	3.24 ± 2.27	2.18 ± 2.02*
% LPI	0.627	0.19 ± 0.09	0.20 ± 0.08	0.18 ± 0.05
% LPS	0.704	0.56 ± 0.29	0.64 ± 0.52	0.50 ± 0.14

Table S4. Univariable and multivariable associations between lipid classes or species and BMI, with or without adjustment for age.
Data presented as β-coefficients and corresponding 95% confidence intervals (CI). All lipids were log-transformed to the base 10 prior to analyses, and all p-values represent significance of associations after correcting for multiple comparisons using the Benjamini-Hochberg method (bold values indicate $p < 0.05$ after correction). Adjusted β: Multivariable models adjusted for age.

BMI						
**Lipid class**	**Unadjusted β (95% CI)**	**p**	**R^2 (p-value)**	**Adjusted β (95% CI)**	**p**	**R^2 (p-value)**
PC	-0.504 (-0.22, -0.79)	**0.012**	0.165 (0.001)	-0.418 (-0.13, -0.71)	0.055	0.213 (0.001)
PE	-0.489 (-0.20, -0.77)	**0.012**	0.157 (0.001)	-0.410 (-0.12, -0.70)	0.055	0.196 (0.001)
PI	-0.386 (-0.09, -0.68)	**0.033**	0.092 (0.013)	-0.309 (-0.01, -0.61)	0.119	0.127 (0.010)
PS	-0.406 (-0.01, -0.81)	0.078	0.050 (0.052)	-0.364 (0.06, -0.78)	0.166	0.041 (0.120)
SM	-0.419 (-0.06, -0.78)	**0.048**	0.070 (0.026)	-0.309 (0.05, -0.67)	0.168	0.127 (0.010)
Cer	-0.530 (-0.19, -0.87)	**0.018**	0.131 (0.003)	-0.46 (-0.11, -0.81)	0.062	0.146 (0.005)
LPC	-0.587 (-0.14, -1.04)	**0.033**	0.090 (0.013)	-0.471 (-0.01, -0.93)	0.119	0.125 (0.010)
LPE	-1.114 (-0.31, -1.92)	**0.029**	0.102 (0.009)	-0.823 (-0.02, -1.62)	0.119	0.185 (0.002)
LPI	-0.454 (0.01, -0.92)	0.091	0.045 (0.062)	-0.392 (0.09, -0.88)	0.188	0.041 (0.119)
LPS	-0.372 (0.16, -0.90)	0.214	0.015 (0.176)	-0.351 (0.21, -0.91)	0.289	-0.002 (0.390)
**Lipid species**	**Unadjusted β (95% CI)**	**p**	**R^2 (p-value)**	**Adjusted β (95% CI)**	**p**	**R^2 (p-value)**
PC 28:0	-0.919 (-0.38, -1.45)	**0.012**	0.166 (0.001)	-0.858 (-0.29, -1.42)	**0.052**	0.157 (0.005)
SM d18:1/20:1	-0.624 (-0.21, -1.04)	**0.024**	0.119 (0.005)	-0.538 (-0.11, -0.97)	0.079	0.134 (0.008)
SM d18:1/22:0	-0.634 (-0.17, -1.10)	**0.029**	0.099 (0.010)	-0.517 (-0.04, -0.99)	0.110	0.131 (0.008)
LPC 20:0	-1.016 (-0.47, -1.57)	**0.011**	0.178 (0.001)	-0.908 (-0.34, -1.48)	**0.052**	0.190 (0.001)
LPC 20:3	-0.559 (-0.10, -1.02)	**0.042**	0.076 (0.021)	-0.478 (0.00, -0.96)	0.120	0.083 (0.036)
LPE 16:0	-0.849 (-0.29, -1.41)	**0.021**	0.123 (0.004)	-0.636 (-0.08, -1.19)	0.096	0.214 (0.001)
LPE 18:1	-0.842 (-0.15, -1.54)	**0.042**	0.077 (0.021)	-0.572 (0.11, -1.25)	0.170	0.179 (0.002)
LPE 20:1	-0.968 (-0.16, -1.78)	**0.044**	0.074 (0.023)	-0.676 (0.13, -1.48)	0.170	0.159 (0.004)
LPE 18:2	-1.004 (-0.25, -1.76)	**0.032**	0.093 (0.012)	-0.716 (0.03, -1.46)	0.130	0.188 (0.001)
LPE 20:2	-0.889 (-0.09, -1.69)	**0.054**	0.063 (0.033)	-0.649 (0.16, -1.45)	0.188	0.119 (0.012)
LPE 20:3	-0.914 (-0.10, -1.73)	**0.054**	0.063 (0.033)	-0.621 (0.19, -1.43)	0.208	0.148 (0.005)
LPE 22:3	-1.178 (-0.25, -2.10)	**0.036**	0.088 (0.016)	-0.853 (0.08, -1.78)	0.141	0.160 (0.004)
LPE 20:4	-1.477 (-0.45, -2.51)	**0.026**	0.109 (0.007)	-1.153 (-0.11, -2.19)	0.105	0.168 (0.003)
LPE 22:4	-1.554 (-0.43, -2.68)	**0.029**	0.101 (0.009)	-1.180 (-0.05, -2.31)	0.116	0.169 (0.002)
LPI 18:0	-0.482 (-0.03, -0.93)	0.063	0.058 (0.040)	-0.440 (0.03, -0.91)	0.134	0.047 (0.102)
LPI 20:4	-0.411 (0.08, -0.90)	0.141	0.030 (0.105)	-0.326 (0.18, -0.83)	0.280	0.037 (0.136)

Table S5. Univariable and multivariable associations between lipid classes or species and CRP with or without adjustment for age.
Data presented as β-coefficients and corresponding 95% confidence intervals (CI). All lipids were log-transformed to the base 10 prior to analyses, and all p-values represent significance of associations after correcting for multiple comparisons using the Benjamini-Hochberg method (bold values indicate $p < 0.05$ after correction). Adjusted β: Multivariable models adjusted for age.

CRP						
**Lipid class**	**Unadjusted β (95% CI)**	**p**	**R^2 (p-value)**	**Adjusted β (95% CI)**	**p**	**R^2 (p-value)**
PC	-0.138 (-0.06, -0.22)	**0.012**	0.160 (0.001)	-0.117 (-0.04, -0.19)	**0.035**	0.250 (>0.001)
PE	-0.138 (-0.06, -0.22)	**0.012**	0.164 (0.001)	-0.118 (-0.04, -0.20)	**0.035**	0.241 (>0.001)
PI	-0.129 (-0.05, -0.21)	**0.015**	0.138 (0.003)	-0.112 (-0.03, -0.19)	**0.040**	0.190 (0.002)
PS	-0.168 (-0.06, -0.28)	**0.017**	0.128 (0.004)	-0.158 (-0.05, -0.27)	**0.040**	0.123 (0.012)
SM	-0.145 (-0.05, -0.24)	**0.019**	0.118 (0.006)	-0.121 (-0.02, -0.22)	**0.050**	0.198 (0.001)
Cer	-0.129 (-0.03, -0.23)	**0.027**	0.096 (0.012)	-0.110 (-0.01, -0.21)	0.068	0.140 (0.007)
LPC	-0.190 (-0.06, -0.32)	**0.017**	0.125 (0.005)	-0.165 (-0.04, -0.29)	**0.044**	0.172 (0.003)
LPE	-0.256 (-0.02, -0.49)	0.059	0.062 (0.037)	-0.191 (0.03, -0.42)	0.141	0.174 (0.003)
LPI	-0.147 (-0.01, -0.28)	0.056	0.064 (0.035)	-0.132 (0.00, -0.27)	0.102	0.066 (0.063)
LPS	-0.133 (0.02, -0.28)	0.112	0.036 (0.089)	-0.129 (0.03, -0.28)	0.150	0.019 (0.228)
**Lipid species**	**Unadjusted β (95% CI)**	**p**	**R^2 (p-value)**	**Adjusted β (95% CI)**	**p**	**R^2 (p-value)**
PC 28:0	-0.137 (0.03, -0.30)	0.130	0.033 (0.108)	-0.109 (0.06, -0.27)	0.241	0.059 (0.086)
SM d18:1/20:1	-0.156 (-0.04, -0.28)	**0.029**	0.092 (0.014)	-0.133 (-0.01, -0.25)	0.073	0.134 (0.009)
SM d18:1/22:0	-0.182 (-0.05, -0.31)	**0.023**	0.106 (0.009)	-0.153 (-0.02, -0.28)	0.059	0.167 (0.003)
LPC 20:0	-0.246 (-0.09, -0.41)	**0.017**	0.131 (0.004)	-0.215 (-0.06, -0.37)	**0.043**	0.175 (0.002)
LPC 20:3	-0.154 (-0.02, -0.29)	**0.047**	0.071 (0.027)	-0.135 (0.00, -0.27)	0.096	0.087 (0.035)
LPE 16:0	-0.214 (-0.05, -0.38)	**0.028**	0.094 (0.013)	-0.167 (-0.01, -0.32)	0.077	0.213 (0.001)
LPE 18:1	-0.232 (-0.03, -0.43)	**0.044**	0.074 (0.025)	-0.174 (0.01, -0.36)	0.114	0.196 (0.001)
LPE 20:1	-0.297 (-0.07, -0.52)	**0.028**	0.093 (0.014)	-0.233 (-0.02, -0.45)	0.078	0.203 (0.001)
LPE 18:2	-0.273 (-0.06, -0.49)	**0.033**	0.086 (0.017)	-0.212 (0.00, -0.42)	0.092	0.201 (0.001)
LPE 20:2	-0.302 (-0.08, -0.53)	**0.025**	0.100 (0.011)	-0.252 (-0.03, -0.47)	0.067	0.165 (0.003)
LPE 20:3	-0.207 (0.03, -0.44)	0.113	0.035 (0.090)	-0.141 (0.08, -0.37)	0.261	0.151 (0.005)
LPE 22:3	-0.214 (0.06, -0.49)	0.153	0.025 (0.132)	-0.137 (0.13, -0.40)	0.341	0.141 (0.008)
LPE 20:4	-0.285 (0.02, -0.59)	0.094	0.042 (0.072)	-0.208 (0.09, -0.50)	0.210	0.133 (0.009)
LPE 22:4	-0.316 (0.01, -0.65)	0.089	0.045 (0.066)	-0.230 (0.09, -0.55)	0.202	0.144 (0.007)
LPI 18:0	-0.155 (-0.03, -0.28)	**0.037**	0.081 (0.020)	-0.145 (-0.01, -0.27)	0.073	0.075 (0.049)
LPI 20:4	-0.148 (-0.01, -0.29)	0.063	0.059 (0.041)	-0.131 (0.01, -0.27)	0.114	0.067 (0.061)

Table S6. **Univariable and multivariable associations between lipid classes or species and HOMA-IR with or without adjustment for age.**
Data presented as β-coefficients and corresponding 95% confidence intervals (CI). All lipids were log-transformed to the base 10 prior to analyses, and all p-values represent significance of associations after correcting for multiple comparisons using the Benjamini-Hochberg method (bold values indicate $p < 0.05$ after correction). Adjusted β: Multivariable models adjusted for age.

HOMA-IR						
**Lipid class**	**Unadjusted β (95% CI)**	**p**	**R^2 (p-value)**	**Adjusted β (95% CI)**	**p**	**R^2 (p-value)**
PC	-0.179 (-0.09, -0.27)	**0.003**	0.202 (>0.001)	-0.153 (-0.06, -0.24)	**0.010**	0.298 (>0.001)
PE	-0.180 (-0.09, -0.27)	**0.003**	0.208 (>0.001)	-0.155 (-0.07, -0.24)	**0.010**	0.296 (>0.001)
PI	-0.161 (-0.07, -0.26)	**0.007**	0.161 (0.002)	-0.139 (-0.05, -0.23)	**0.021**	0.228 (0.001)
PS	-0.168 (-0.04, -0.30)	**0.027**	0.091 (0.015)	-0.153 (-0.02, -0.29)	0.058	0.093 (0.031)
SM	-0.164 (-0.05, -0.28)	**0.019**	0.110 (0.008)	-0.134 (-0.02, -0.25)	**0.054**	0.198 (0.001)
Cer	-0.203 (-0.09, -0.31)	**0.004**	0.189 (0.001)	-0.181 (-0.07, -0.29)	**0.011**	0.228 (0.001)
LPC	-0.180 (-0.03, -0.33)	**0.039**	0.076 (0.025)	-0.149 (0.00, -0.30)	0.091	0.123 (0.013)
LPE	-0.316 (-0.05, -0.59)	**0.040**	0.074 (0.026)	-0.248 (0.02, -0.51)	0.099	0.158 (0.005)
LPI	-0.169 (-0.01, -0.32)	**0.053**	0.063 (0.038)	-0.148 (0.01, -0.31)	0.099	0.073 (0.054)
LPS	-0.169 (0.01, -0.35)	0.082	0.045 (0.066)	-0.162 (0.02, -0.34)	0.115	0.029 (0.176)
**Lipid species**	**Unadjusted β (95% CI)**	**p**	**R^2 (p-value)**	**Adjusted β (95% CI)**	**p**	**R^2 (p-value)**
PC 28:0	-0.401 (-0.24, -0.56)	**0.001**	0.316 (>0.001)	-0.381 (-0.21, -0.55)	**0.003**	0.313 (>0.001)
SM d18:1/20:1	-0.244 (-0.11, -0.38)	**0.005**	0.18 (0.001)	-0.217 (-0.08, -0.35)	**0.013**	0.219 (0.001)
SM d18:1/22:0	-0.236 (-0.09, -0.39)	**0.011**	0.137 (0.003)	-0.204 (-0.05, -0.35)	**0.033**	0.190 (0.002)
LPC 20:0	-0.346 (-0.17, -0.53)	**0.003**	0.200 (>0.001)	-0.313 (-0.13, -0.49)	**0.010**	0.229 (>0.001)
LPC 20:3	-0.198 (-0.04, -0.35)	**0.027**	0.092 (0.015)	-0.176 (-0.02, -0.33)	0.060	0.106 (0.021)
LPE 16:0	-0.267 (-0.08, -0.46)	**0.018**	0.113 (0.007)	-0.215 (-0.03, -0.40)	**0.052**	0.214 (0.001)
LPE 18:1	-0.228 (0.00, -0.46)	0.077	0.048 (0.060)	-0.165 (0.06, -0.39)	0.187	0.152 (0.006)
LPE 20:1	-0.338 (-0.07, -0.60)	**0.029**	0.089 (0.016)	-0.268 (-0.01, -0.53)	0.078	0.181 (0.002)
LPE 18:2	-0.322 (-0.07, -0.57)	**0.026**	0.093 (0.014)	-0.258 (-0.02, -0.50)	0.073	0.180 (0.002)
LPE 20:2	-0.317 (-0.05, -0.58)	**0.038**	0.078 (0.023)	-0.259 (0.00, -0.52)	0.090	0.137 (0.009)
LPE 20:3	-0.319 (-0.05, -0.59)	**0.039**	0.075 (0.025)	-0.248 (0.02, -0.51)	0.099	0.170 (0.003)
LPE 22:3	-0.393 (-0.09, -0.70)	**0.027**	0.096 (0.015)	-0.312 (-0.02, -0.61)	0.075	0.183 (0.003)
LPE 20:4	-0.419 (-0.07, -0.76)	**0.035**	0.081 (0.021)	-0.344 (0.00, -0.68)	0.086	0.138 (0.008)
LPE 22:4	-0.389 (-0.01, -0.77)	0.065	0.054 (0.049)	-0.301 (0.07, -0.67)	0.149	0.124 (0.013)
LPI 18:0	-0.178 (-0.03, -0.33)	**0.038**	0.078 (0.023)	-0.163 (-0.01, -0.32)	0.073	0.077 (0.049)
LPI 20:4	-0.139 (0.03, -0.30)	0.117	0.032 (0.103)	-0.114 (0.05, -0.28)	0.209	0.052 (0.097)

Table S7. **Univariable and multivariable associations between lipid classes or species and fasting insulin with or without adjustment for age.**
Data presented as β-coefficients and corresponding 95% confidence intervals (CI). All lipids were log-transformed to the base 10 prior to analyses, and all p-values represent significance of associations after correcting for multiple comparisons using the Benjamini-Hochberg method (bold values indicate $p < 0.05$ after correction). Adjusted β: Multivariable models adjusted for age.

**Fasting insulin**						
**Lipid class**	**Unadjusted β (95% CI)**	**p**	**R^2 (p-value)**	**Adjusted β (95% CI)**	**p**	**R^2 (p-value)**
PC	-0.198 (-0.09, -0.30)	**0.005**	0.189 (0.001)	-0.175 (-0.08, -0.27)	**0.009**	0.303 (>0.001)
PE	-0.198 (-0.09, -0.30)	**0.005**	0.191 (0.001)	-0.176 (-0.08, -0.27)	**0.009**	0.298 (>0.001)
PI	-0.180 (-0.07, -0.29)	**0.009**	0.154 (0.002)	-0.160 (-0.06, -0.26)	**0.018**	0.235 (>0.001)
PS	-0.186 (-0.04, -0.34)	**0.035**	0.085 (0.019)	-0.173 (-0.02, -0.32)	0.058	0.093 (0.031)
SM	-0.177 (-0.04, -0.31)	**0.027**	0.097 (0.013)	-0.151 (-0.02, -0.28)	**0.052**	0.198 (0.001)
Cer	-0.214 (-0.09, -0.34)	**0.008**	0.159 (0.002)	-0.195 (-0.07, -0.32)	**0.016**	0.213 (0.001)
LPC	-0.195 (-0.02, -0.37)	**0.052**	0.067 (0.033)	-0.169 (0.00, -0.34)	0.091	0.124 (0.013)
LPE	-0.341 (-0.03, -0.65)	0.055	0.065 (0.035)	-0.283 (0.01, -0.58)	0.100	0.160 (0.004)
LPI	-0.183 (-0.01, -0.36)	0.068	0.055 (0.048)	-0.165 (0.01, -0.34)	0.111	0.071 (0.057)
LPS	-0.181 (0.02, -0.38)	0.105	0.038 (0.085)	-0.173 (0.03, -0.38)	0.133	0.023 (0.205)
**Lipid species**	**Unadjusted β (95% CI)**	**p**	**R^2 (p-value)**	**Adjusted β (95% CI)**	**p**	**R^2 (p-value)**
PC 28:0	-0.426 (-0.23, -0.62)	**0.003**	0.262 (>0.001)	-0.401 (-0.20, -0.60)	**0.006**	0.269 (>0.001)
SM d18:1/20:1	-0.264 (-0.11, -0.42)	**0.008**	0.161 (0.002)	-0.240 (-0.09, -0.39)	**0.016**	0.212 (0.001)
SM d18:1/22:0	-0.247 (-0.07, -0.42)	**0.022**	0.112 (0.008)	-0.218 (-0.05, -0.39)	**0.040**	0.179 (0.002)
LPC 20:0	-0.366 (-0.16, -0.57)	**0.007**	0.170 (0.001)	-0.336 (-0.13, -0.54)	**0.013**	0.213 (0.001)
LPC 20:3	-0.220 (-0.04, -0.40)	**0.034**	0.086 (0.018)	-0.200 (-0.02, -0.38)	0.058	0.107 (0.021)
LPE 16:0	-0.291 (-0.08, -0.51)	**0.024**	0.102 (0.011)	-0.247 (-0.04, -0.45)	**0.047**	0.217 (0.001)
LPE 18:1	-0.241 (0.03, -0.51)	0.103	0.039 (0.083)	-0.188 (0.07, -0.44)	0.181	0.153 (0.005)
LPE 20:1	-0.365 (-0.06, -0.67)	**0.04**	0.079 (0.023)	-0.306 (-0.02, -0.60)	0.075	0.182 (0.002)
LPE 18:2	-0.354 (-0.07, -0.64)	**0.035**	0.085 (0.019)	-0.299 (-0.03, -0.57)	0.066	0.184 (0.002)
LPE 20:2	-0.314 (-0.01, -0.62)	0.068	0.055 (0.049)	-0.264 (0.03, -0.56)	0.118	0.125 (0.012)
LPE 20:3	-0.327 (-0.02, -0.64)	0.065	0.057 (0.045)	-0.266 (0.03, -0.56)	0.118	0.165 (0.004)
LPE 22:3	-0.428 (-0.08, -0.78)	**0.037**	0.085 (0.020)	-0.356 (-0.02, -0.69)	0.073	0.185 (0.003)
LPE 20:4	-0.453 (-0.06, -0.85)	**0.048**	0.071 (0.029)	-0.389 (0.00, -0.77)	0.086	0.139 (0.008)
LPE 22:4	-0.420 (0.01, -0.85)	0.085	0.047 (0.063)	-0.345 (0.07, -0.77)	0.142	0.126 (0.012)
LPI 18:0	-0.194 (-0.02, -0.37)	**0.049**	0.070 (0.030)	-0.180 (-0.01, -0.35)	0.077	0.074 (0.053)
LPI 20:4	-0.154 (0.03, -0.34)	0.131	0.029 (0.113)	-0.133 (0.05, -0.32)	0.197	0.054 (0.091)

Table S8. **Univariable and multivariable associations between lipid species or classes and Fasting glucose with or without adjustment for age.**
Data presented as β-coefficients and corresponding 95% confidence intervals (CI). All lipids were log-transformed to the base 10 prior to analyses, and all p-values represent significance of associations after correcting for multiple comparisons using the Benjamini-Hochberg method (bold values indicate $p < 0.05$ after correction). Adjusted β: Multivariable models adjusted for age.

Fasting glucose						
**Lipid class**	**Unadjusted β (95% CI)**	**p**	**R^2 (p-value)**	**Adjusted β (95% CI)**	**p**	**R^2 (p-value)**
PC	-0.518 (-0.09, -0.95)	0.090	0.076 (0.021)	-0.350 (0.09, -0.79)	0.337	0.133 (0.008)
PE	-0.527 (-0.10, -0.95)	0.090	0.081 (0.018)	-0.375 (0.07, -0.82)	0.316	0.125 (0.010)
PI	-0.417 (0.01, -0.85)	0.120	0.044 (0.063)	-0.270 (0.18, -0.72)	0.414	0.084 (0.035)
PS	-0.465 (0.12, -1.05)	0.179	0.025 (0.123)	-0.385 (0.24, -1.01)	0.405	0.017 (0.237)
SM	-0.557 (-0.04, -1.08)	0.095	0.058 (0.040)	-0.361 (0.18, -0.90)	0.391	0.110 (0.016)
Cer	-0.750 (-0.26, -1.24)	0.061	0.127 (0.004)	-0.639 (-0.12, -1.15)	0.288	0.135 (0.007)
LPC	-0.600 (0.06, -1.26)	0.138	0.037 (0.081)	-0.372 (0.32, -1.06)	0.465	0.079 (0.041)
LPE	-1.155 (0.04, -2.35)	0.120	0.045 (0.062)	-0.593 (0.61, -1.80)	0.488	0.138 (0.007)
LPI	-0.492 (0.18, -1.17)	0.209	0.018 (0.159)	-0.373 (0.35, -1.10)	0.465	0.016 (0.244)
LPS	-0.528 (0.23, -1.29)	0.221	0.015 (0.180)	-0.502 (0.32, -1.32)	0.408	-0.003 (0.404)
**Lipid species**	**Unadjusted β (95% CI)**	**p**	**R^2 (p-value)**	**Adjusted β (95% CI)**	**p**	**R^2 (p-value)**
PC 28:0	-1.586 (-0.87, -2.30)	**0.015**	0.258 (>0.001)	-1.557 (-0.78, -2.33)	**0.054**	0.243 (>0.001)
SM d18:1/20:1	-0.809 (-0.20, -1.42)	0.084	0.094 (0.012)	-0.661 (-0.02, -1.3)	0.304	0.106 (0.018)
SM d18:1/22:0	-0.941 (-0.28, -1.60)	0.079	0.108 (0.007)	-0.750 (-0.05, -1.45)	0.304	0.130 (0.009)
LPC 20:0	-1.263 (-0.45, -2.07)	0.061	0.130 (0.003)	-1.073 (-0.22, -1.93)	0.288	0.139 (0.007)
LPC 20:3	-0.582 (0.09, -1.26)	0.158	0.032 (0.097)	-0.423 (0.29, -1.14)	0.422	0.042 (0.118)
LPE 16:0	-0.897 (-0.07, -1.73)	0.095	0.059 (0.039)	-0.489 (0.35, -1.32)	0.426	0.160 (0.003)
LPE 18:1	-0.932 (0.08, -1.95)	0.132	0.039 (0.077)	-0.421 (0.60, -1.44)	0.533	0.148 (0.005)
LPE 20:1	-1.170 (0.01, -2.35)	0.116	0.048 (0.056)	-0.632 (0.56, -1.83)	0.465	0.134 (0.008)
LPE 18:2	-1.076 (0.04, -2.19)	0.120	0.044 (0.064)	-0.525 (0.60, -1.65)	0.504	0.148 (0.005)
LPE 20:2	-1.476 (-0.35, -2.60)	0.085	0.092 (0.013)	-1.088 (0.08, -2.26)	0.309	0.131 (0.008)
LPE 20:3	-1.423 (-0.26, -2.59)	0.090	0.078 (0.020)	-0.925 (0.27, -2.12)	0.340	0.150 (0.005)
LPE 22:3	-1.435 (-0.09, -2.78)	0.096	0.059 (0.042)	-0.874 (0.50, -2.25)	0.396	0.134 (0.009)
LPE 20:4	-1.513 (0.02, -3.04)	0.116	0.047 (0.058)	-0.879 (0.69, -2.45)	0.448	0.115 (0.014)
LPE 22:4	-1.422 (0.25, -3.10)	0.162	0.031 (0.102)	-0.671 (1.03, -2.38)	0.538	0.114 (0.014)
LPI 18:0	-0.506 (0.15, -1.16)	0.190	0.023 (0.136)	-0.421 (0.28, -1.12)	0.414	0.013 (0.263)
LPI 20:4	-0.341 (0.37, -1.05)	0.389	-0.002 (0.352)	-0.163 (0.59, -0.92)	0.732	0.012 (0.272)

Table S9. Univariable and multivariable associations between lipid classes or species and HbA1c, with or without adjustment for age.
Data presented as β-coefficients and corresponding 95% confidence intervals (CI). All lipids were log-transformed to the base 10 prior to analyses, and all p-values represent significance of associations after correcting for multiple comparisons using the Benjamini-Hochberg method (bold values indicate $p < 0.05$ after correction). Adjusted β: Multivariable models adjusted for age.

HbA1c						
**Lipid class**	**Unadjusted β (95% CI)**	**p**	**R^2 (p-value)**	**Adjusted β (95% CI)**	**p**	**R^2 (p-value)**
PC	-0.968 (-0.28, -1.66)	**0.044**	0.105 (0.008)	-0.731 (-0.03, -1.43)	0.180	0.159 (0.003)
PE	-0.967 (-0.28, -1.65)	**0.044**	0.107 (0.008)	-0.750 (-0.05, -1.45)	0.180	0.150 (0.005)
PI	-0.706 (0.00, -1.41)	0.093	0.049 (0.054)	-0.491 (0.23, -1.21)	0.306	0.091 (0.029)
PS	-0.799 (0.15, -1.75)	0.143	0.030 (0.104)	-0.680 (0.32, -1.68)	0.306	0.022 (0.205)
SM	-1.103 (-0.27, -1.93)	**0.051**	0.094 (0.012)	-0.833 (0.02, -1.68)	0.186	0.140 (0.006)
Cer	-1.250 (-0.46, -2.04)	**0.037**	0.133 (0.003)	-1.079 (-0.25, -1.91)	0.144	0.144 (0.006)
LPC	-1.062 (0.01, -2.14)	0.094	0.047 (0.058)	-0.733 (0.38, -1.84)	0.317	0.088 (0.031)
LPE	-2.134 (-0.21, -4.06)	0.071	0.062 (0.034)	-1.333 (0.59, -3.26)	0.305	0.152 (0.004)
LPI	-0.663 (0.45, -1.77)	0.291	0.006 (0.248)	-0.467 (0.70, -1.64)	0.508	0.008 (0.299)
LPS	-0.746 (0.50, -2.00)	0.291	0.006 (0.248)	-0.686 (0.64, -2.01)	0.402	-0.010 (0.495)
**Lipid species**	**Unadjusted β (95% CI)**	**p**	**R^2 (p-value)**	**Adjusted β (95% CI)**	**p**	**R^2 (p-value)**
PC 28:0	-2.444 (-1.28, -3.61)	**0.035**	0.233 (>0.001)	-2.342 (-1.10, -3.58)	0.123	0.222 (0.001)
SM d18:1/20:1	-1.57 (-0.60, -2.54)	**0.037**	0.140 (0.002)	-1.368 (-0.36, -2.38)	0.144	0.149 (0.005)
SM d18:1/22:0	-1.728 (-0.67, -2.79)	**0.037**	0.141 (0.002)	-1.458 (-0.36, -2.56)	0.144	0.162 (0.003)
LPC 20:0	-2.221 (-0.92, -3.52)	**0.037**	0.154 (0.002)	-1.942 (-0.58, -3.30)	0.144	0.164 (0.003)
LPC 20:3	-1.081 (0.02, -2.18)	0.094	0.047 (0.059)	-0.856 (0.29, -2.00)	0.272	0.055 (0.080)
LPE 16:0	-1.649 (-0.31, -2.99)	0.056	0.079 (0.019)	-1.067 (0.27, -2.40)	0.239	0.177 (0.002)
LPE 18:1	-1.686 (-0.04, -3.33)	0.088	0.052 (0.049)	-0.954 (0.68, -2.58)	0.357	0.158 (0.004)
LPE 20:1	-1.864 (0.06, -3.79)	0.098	0.044 (0.063)	-1.065 (0.86, -2.99)	0.374	0.135 (0.007)
LPE 18:2	-1.996 (-0.19, -3.80)	0.071	0.062 (0.034)	-1.212 (0.58, -3.00)	0.306	0.162 (0.003)
LPE 20:2	-2.389 (-0.55, -4.22)	**0.051**	0.090 (0.014)	-1.802 (0.08, -3.69)	0.191	0.134 (0.008)
LPE 20:3	-2.423 (-0.53, -4.31)	**0.052**	0.087 (0.015)	-1.692 (0.22, -3.60)	0.210	0.160 (0.003)
LPE 22:3	-2.333 (-0.13, -4.54)	0.080	0.058 (0.043)	-1.498 (0.72, -3.71)	0.306	0.137 (0.008)
LPE 20:4	-2.676 (-0.20, -5.16)	0.077	0.058 (0.039)	-1.762 (0.75, -4.28)	0.300	0.126 (0.010)
LPE 22:4	-2.587 (0.13, -5.30)	0.103	0.042 (0.067)	-1.513 (1.22, -4.25)	0.374	0.124 (0.011)
LPI 18:0	-0.710 (0.36, -1.79)	0.246	0.012 (0.200)	-0.567 (0.57, -1.70)	0.419	0.005 (0.325)
LPI 20:4	-0.469 (0.70, -1.63)	0.470	-0.007 (0.434)	-0.198 (1.02, -1.41)	0.777	0.010 (0.283)
